# Author response to: Comment on: Outcomes after totally minimally invasive *versus* hybrid and open Ivor Lewis oesophagectomy: results from the International Esodata Study Group

**DOI:** 10.1093/bjs/znac338

**Published:** 2022-10-18

**Authors:** Berend J van der Wilk, Sjoerd M Lagarde, Mark I van Berge Henegouwen

**Affiliations:** Department of Surgical Oncology and Gastrointestinal Surgery, Erasmus MC Cancer Institute, Rotterdam, the Netherlands; Department of Surgical Oncology and Gastrointestinal Surgery, Erasmus MC Cancer Institute, Rotterdam, the Netherlands; Department of Surgery, Amsterdam University Medical Centre, University of Amsterdam, Amsterdam Cancer Centre, Amsterdam, the Netherlands

*Dear Editor*

We thank the authors for their comments on our study^1^. We would be happy to elaborate further on the questions raised by the authors.

We agree with the authors that additional variables would be interesting to take into account for the primary outcome. In a statistical analysis, however, the number of confounders that can be included is limited. We therefore deliberately limited the number of confounders to those described to have the greatest potential effect on the primary and secondary outcomes.

The authors point out that there were differences in baseline characteristics in terms of preoperative treatment, tumour location, and clinical nodal status. We included these possible confounders for the outcomes in our multilevel mixed statistical model. It could, therefore, be concluded that surgical approach was an independent factor associated with pneumonia, despite these differences in baseline characteristics.

Finally, we believe that a proficiency gain curve could have an effect on our results. As the data were anonymized, they were not sufficient to correlate the proficiency gain curve with postoperative outcomes. It should be noted, however, that all participating hospitals were high-volume centres and that the most experienced centres did not have the lowest anastomotic leakage rates *per se*.
